# Measuring brain perfusion by CT or MR as ancillary tests for diagnosis of brain death: a systematic review and meta-analysis

**DOI:** 10.1093/bjro/tzae037

**Published:** 2024-11-04

**Authors:** João N Ramos, Catarina Pinto, Vera Cruz e Silva, Constantin-Cristian Topriceanu, Sotirios Bisdas

**Affiliations:** Department of Neuroradiology, Centro Hospitalar de Lisboa Ocidental, Lisboa, 1349-019, Portugal; Lysholm Department of Neuroradiology, The National Hospital for Neurology and Neurosurgery, University College London Hospitals NHS Foundation Trust, London WC1N 3BG, United Kingdom; Department of Neuroradiology, Centro Hospitalar Universitário do Porto, Porto, 4050-342, Portugal; Medical Image Analysis Center (MIAC AG), Basel, 4051, Switzerland; Institute of Cardiovascular Science, University College London, London, WC1E 6DD, United Kingdom; Department of Brain Repair and Rehabilitation, Queen Square Institute of Neurology, University College London, London, WC1N 3BG, United Kingdom; Lysholm Department of Neuroradiology, The National Hospital for Neurology and Neurosurgery, University College London Hospitals NHS Foundation Trust, London WC1N 3BG, United Kingdom; Department of Brain Repair and Rehabilitation, Queen Square Institute of Neurology, University College London, London, WC1N 3BG, United Kingdom

**Keywords:** brain death, perfusion, CT, MR, arterial spin labelling

## Abstract

**Objectives:**

To gather and synthesize evidence regarding diagnostic accuracy of perfusion imaging by CT (CTP) or MR (MRP) for brain death (BD) diagnosis.

**Methods:**

A systematic review and meta-analysis was prospectively registered with PROSPERO (CRD42022336353) and conducted in accordance with the PRISMA guidelines and independently by 3 reviewers. PubMed/MEDLINE, EMBASE and Cochrane Database were searched for relevant studies. Quality Assessment of Diagnostic Accuracy Studies-2 was used to assess studies’ quality. Meta-analysis was performed using univariate random-effects models.

**Results:**

Ten studies (328 patients) were included. Perfusion imaging (most commonly CTP, *n* = 8 studies) demonstrated a high sensitivity of 96.1% (95% CI, 89.5-98.6) for BD, consistent in subgroup analysis at 95.5% (95% CI, 86.5-98.6). Unfortunately, it was not feasible to calculate other metrics. Additionally, evidence of publication bias was identified in our findings.

**Conclusions:**

The sensitivity of CTP or MRP for BD diagnosis is very high, comparable to CTA and TCD. However, considering most studies were retrospective, and lacked control groups and unambiguous criteria for perfusion imaging in BD assessment, results should be interpreted with caution. Future studies, ideally prospective, multi-centre, and with control groups are of utmost importance for validation of these methods, particularly with standardized technical parameters.

**Advances in knowledge:**

Cerebral perfusion imaging using CT or MRI demonstrates high sensitivity in diagnosing BD, on par with CTA and TCD. Recommended by the World Brain Death group, this method holds promise for further investigation in this area.

**PROSPERO registration number:**

CRD42022336353

## Introduction

Brain death (BD) has recently garnered attention in numerous reviews, consensus statements, and international guidelines^1–4^ due to its significant implications for neuroprognostication, socio-ethical consequences, and potential organ donation. The neurological exam, particularly tailored to this scenario, holds paramount importance in establishing the criteria for BD. The primary objective of the neurological assessment is to determine the complete absence of brain function, specifically the brainstem reflexes, which is deemed irreversible. Neuroimaging, predominantly by structural CT or MRI, serves a crucial role in ruling out alternative diagnoses and documenting probable irreversible injury hence. Therefore, imaging results can be pivotal in confirming BD. Neuroimaging is often utilized when challenges arise in meeting the minimum clinical criteria for BD due to confounding factors or limitations in the clinical evaluation process. Digital subtraction angiography is considered the gold standard, while radionuclide imaging or transcranial Doppler (TCD) are viewed as viable alternatives.^1^ Although CT angiography (CTA) has shown promising results as an ancillary test and found its way into clinical practice,^5–7^ its validation is still pending.^8^

Conversely, perfusion imaging using CT (CTP) or MR (MRP) is increasingly being utilized in various clinical settings such as acute stroke assessment^9^^,^^10^ and neuro-oncology,^11^^,^^12^ necessitating broader availability of CTP and MRP equipment and enhanced training for clinicians worldwide. While recent BD guidelines acknowledge the potential utility of CTP in these contexts, its definitive validation is awaited.^1^ To address the uncertainty surrounding the utility of perfusion imaging in determining BD, our aim is to consolidate existing evidence and evaluate the role of CTP/MRP as supplementary tests in the diagnosis of BD.

## Methods

The protocol was registered in the International Prospective Register of Systematic Reviews, PROSPERO (CRD42022336353). The authors consulted the Preferred Reporting Items for a Systematic Review and Meta-analysis of Diagnostic Test Accuracy Studies (PRISMA-DTA) to conduct this analysis.^13^

### Search strategy and selection criteria

A systematic search was performed on PubMed/MEDLINE, Cochrane Database, and EMBASE (via Ovid) Library on November 15, 2023, to identify peer-reviewed publications, with no limitations as to publication date or language other than Japanese and Chinese. The full syntax is available in Appendix S1.

Eligible papers were considered regardless of the patients’ age. Studies were excluded if they were non-original (ie, reviews, lecture notes, book chapters, commentaries, erratum, editorial, and conference abstracts) or animal studies. Duplicates were removed using a bibliography manager (Mendeley Desktop v1.19.8).

Three reviewers (J.N.R., C.P., and V.C.S.) independently conducted title screening and abstract screening. Following each stage, a consensus meeting was held to finalize the list of papers for the subsequent stage. During the full-text eligibility phase, the 3 reviewers independently conducted a cross-study review to remove duplicate reports of the same cohorts, prioritizing the most recent or comprehensive cohort. Furthermore, reference lists of included articles were examined to identify additional eligible publications. A review flowchart was developed in accordance with guidelines from the PRISMA group.^14^

### Data extraction

Three reviewers (J.N.R., C.P., and V.C.S.) autonomously gathered data concerning participant demographics (such as the number of individuals in each diagnostic category, age, gender), neuroimaging information (including imaging technique, specifics of contrast administration or acquisition time delay, technical aspects of acquisition and protocol), and precision findings (such as sensitivity, specificity, positive predictive value [PPV], negative predictive value [NPV]) for each outcome—in cases where multiple outcomes were documented.

### Quality assessments

Patient selection, conduct of the study, and interpretation of BD status classification were examined in accordance with established guidelines and consensus statements to address the research question and mitigate potential bias. The quality of this systematic review was ensured by minimizing the risk of bias and concerns regarding applicability following the Quality Assessment of Diagnostic Accuracy Studies-2 (QUADAS-2) questionnaire.^15^ This assessment was independently carried out by 3 authors (J.N.R., C.P., and V.C.S.), with any discrepancies resolved through consensus. In cases where consensus could not be reached, the final decision was made by the principal investigator (S.B.).

### Statistical analysis

In this review, we investigated the primary research question using the pooled data and separately for perfusion imaging done by CT or MR. The analysis was planned to determine the true positive (TP), true negative (TN), false positive (FP), and false negative (FN) rates as part of the quantitative assessment. Univariate random-effects meta-analyses were considered to compute the pooled sensitivity, specificity, PPV, and NPV along with their corresponding 95% CIs.

Assessment of inter-study heterogeneity was performed using Cochran’s *Q* and Higgins *I*^2^ statistics. A Cochran’s *Q* test with a *P*-value <0.05 or *I*^2^ > 50% were interpreted as potentially indicating heterogeneity. Furthermore, publication bias was evaluated through visual inspection of contour-enhanced funnel plots and Egger’s test. An asymmetrical funnel plot or a significant Egger’s test *P*-value suggested the possibility of publication bias.

In small studies, minor variations in TP, TN, FP, and FN values could result in significant changes in sensitivity, specificity, PPV, and NPV. To validate the findings, the meta-analyses were replicated, including only studies with a participant count exceeding 30.

Statistical analysis was carried out using R v4.2.1 (R Foundation for Statistical Computing, Vienna, Austria) using the “meta” and “mada” packages, considering a significance level of *P* < 0.05.

## Results

### Search results

**Figure 1.**
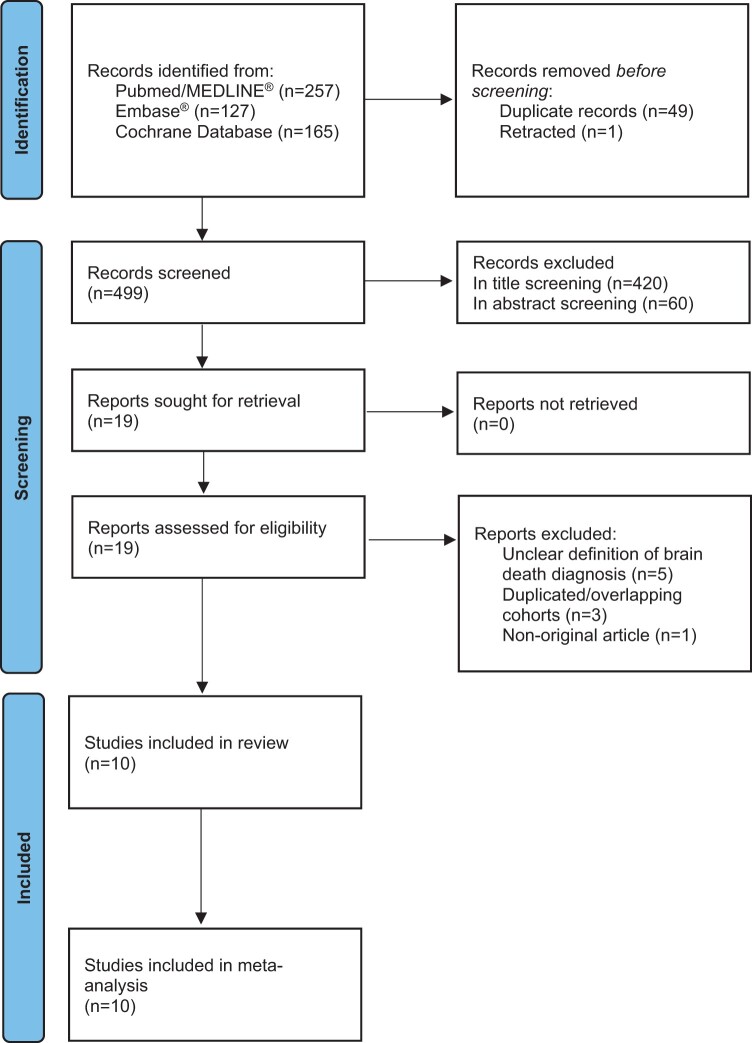
Flowchart of study selection across all stages.

The literature review resulted in the identification of 549 articles. Following the removal of duplicates and retracted studies, 499 articles remained, with 79 passing the initial title screening. Subsequently, 60 articles were excluded based on the inclusion and exclusion criteria during the abstract screening process. Upon conducting a detailed examination of the full texts, 10 articles were deemed suitable for inclusion^16–25^ were included. For a comprehensive visual representation, please refer to Figure 1 for the flowchart and Table 1 for an overview of study characteristics.

**Table 1.** Characteristics of the studies included.

Study year, name (country)	Study type	Study design	Patients (BD), *n*	Females, *n*	Age (years)	Brain death reference standard	Perfusion imaging type	Brain covered (cm)	ROI for AIF	ROI for analysis	Perfusion analysis	Perfusion outcome
2009, Escudero et al (Spain)	Prospective	Case series	27	10	49.7 ± 16.8	Clinical + EEG	CTP	3.2	Manual (MCA)	N/A	Qualitative	Software incapable of automatic postprocessing, “indicating null CBF, CBV, and MTT”
2010, Bohatyrewicz et al (Poland)	Prospective	Case series	24	11	48.0 ± 15.1^a^	Clinical	CTP	Not reported	Not reported	Not reported	Quantitative	Not reported
2013, Shankar et al (Canada)	Retrospective	Case series	11	5	48.2 ± 14.3	National Guidelines	CTP	9.6	Automated	Brainstem only	Qualitative	Matched decrease in CBF and CBV
2013, Sawicki et al (Poland)	Retrospective	Case-control	30	12	53.7 ± 16.4	Clinical	CTP	2.88	Manual (superficial temporal artery and distal MCA)	Supratentorial	Quantitative	Not reported
2015, Kang et al (South Korea)	Retrospective	Case series	5	2	60.8 ± 12.1	Clinical + EEG	MRI-ASL	Not reported	N/A	Not reported	Qualitative	Combination of image findings suggestive of marked brain hypoperfusion, intracranial flow stagnation and patent external carotid circulation
2018, Sawicki et al (Poland)	Prospective	Case-control	50	27	55 ± 18	Clinical	CTP	9.6	Manual (cavernous ICA)	Supra- and infratentorial	Quantitative	CBF < 10, CBV < 1
2018, MacDonald et al (Canada)	Retrospective	Case series	39	Not reported	Not reported	Clinical	CTP	9.6	Not reported	Not reported	Qualitative	Not reported
2020, Yildirim (Turkey)	Prospective	Cohort	61	24	55.1 ± 2.2	Clinical	MRI-DSC	10	Not reported	Not reported	Quantitative	3.5% or more of decrease in the signal
2020, Akdogan et al (Turkey)	Retrospective	Case-control	77	36	43.84 ± 23.25	Clinical	CTP	Not reported	Automated	Not reported	Qualitative	“Complete absence of perfusion in the cerebral parenchyma”
2022, Wang et al (China)	Prospective	Case series	5	2	5.2 ± 2.0^a^	National Guidelines	CTP	16	Not reported	Supra- and infratentorial	Quantitative	CBF < 15, CBV < 1

AIF = arterial input function; ASL = arterial spin labelling; BD = brain death; CBF = cerebral blood flow; CBV = cerebral blood volume; CTP = CT perfusion; DSC = dynamic susceptibility contrast; EEG = electroencephalogram; ICA = internal carotid artery; MCA = middle cerebral artery; MTT = mean transit time; ROI = region of interest.

a Calculated from the available data from the article.

All the included studies, spanning the years 2009 to 2022, were conducted as single-centre investigations in Europe, North America, and Asia. These studies collectively provided insights into BD determination, aligning with local guidelines and international consensus without any instances of clinical misdiagnosis. The total study population comprised 329 patients diagnosed with BD, 129 of them being female based on available data. While most of the studies were retrospective and focused on the BD group, some also featured a control group, totalling 138 patients, with 68 of them being female (49.3%).

The predominant brain perfusion imaging modality under investigation was CTP (*n* = 8), followed by MRP (*n* = 2), ASL (*n* = 1), and DSC (*n* = 1). For further details on study characteristics, including the first author’s name and year, study centre or location, study type, definition of BD, imaging modalities used, average participant age, and sex ratio, please refer to Table 1.

### Qualitative assessment

**Figure 2.**
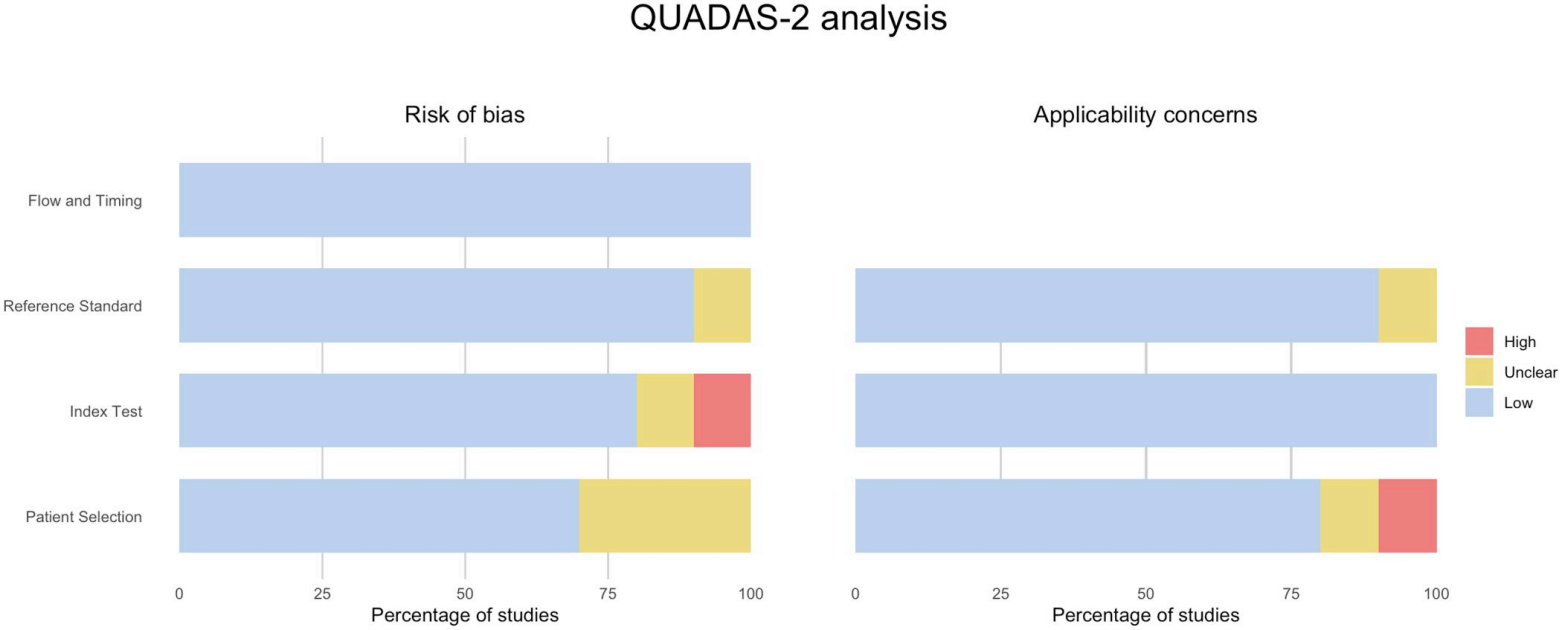
Quality assessment of studies included using QUADAS-2.

The studies included in the analysis demonstrated good methodological quality, as indicated in Figure 2. Evaluation using QUADAS-2 assessed bias risk across 4 domains: patient selection, index test, reference standard, and flow and timing. In terms of patient selection, 30% of the studies were deemed to have an unclear risk of bias due to uncertainty regarding whether the patient sample was consecutive or convenience cohorts. One study raised concerns for applicability due to the absence of criteria mentioned for determining BD. While no issues were identified for flow and timing, one study was found to have a high risk of bias due to insufficient information about perfusion post-processing. In one study related to the reference standard, insufficient reporting of BD criteria led to ambiguity regarding the generalizability of the results and the suitability of the study for inclusion in the current analysis.

### Meta-analysis

**Figure 3.**
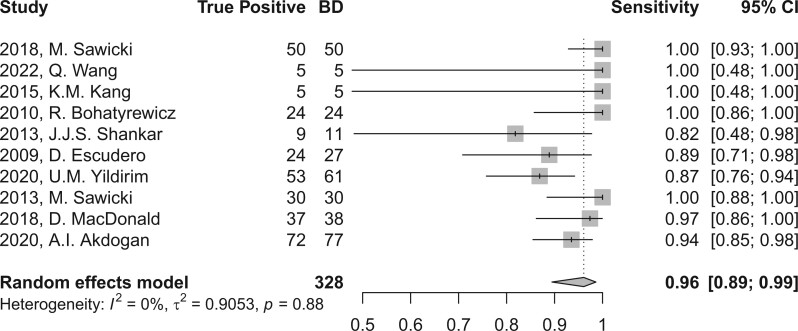
Forest plot of sensitivity of perfusion imaging by CT or MR in BD diagnosis across all studies included and after applying univariate random-effect model.

**Figure 4.**
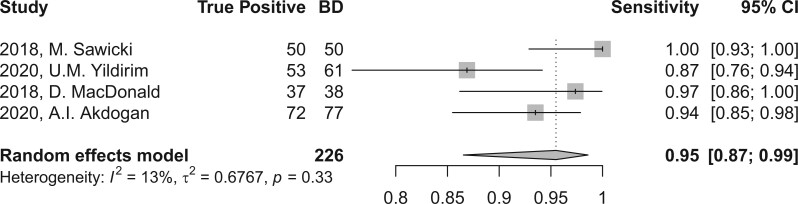
Forest plot of sensitivity sub-analysis using studies including at least 30 patients.

In the evaluation of BD, perfusion brain imaging through CT or MR scans demonstrated a high sensitivity, yielding a combined sensitivity of 96.1 (95% CI, 89.5-98.6) as depicted in Figure 3, and 95.5 (95% CI, 86.5-98.6) when considering studies with sample sizes exceeding 30 subjects, as illustrated in Figure 4. The level of heterogeneity was minimal, with *I*^2^ values of 0.0% and 13.4% (*P* > 0.05), respectively, as shown in Table 2. Due to the absence of control groups and incomplete reporting of TN and FP rates, it was not feasible to aggregate other diagnostic accuracy parameters, such as specificity.

**Table 2.** Summary of the meta-analysis metrics.

Number of studies	Number of participants		Parameter	Heterogeneity		Effect size	Egger’s test
	TP	BD		*I* ^2^	*P*	Pooled estimate (95% CI)	*P*
10	309	328	Sensitivity	0.0%	0.88	96.1 (89.5-98.6)	0.05
4^a^	212	226	Sensitivity	13.4%	0.33	95.5 (86.5-98.6)	^b^

BD = brain death; TP = true positive.

a Those with sample sizes over 30.

b Impossible to calculate Egger’s test given the low number of studies.

**Figure 5.**
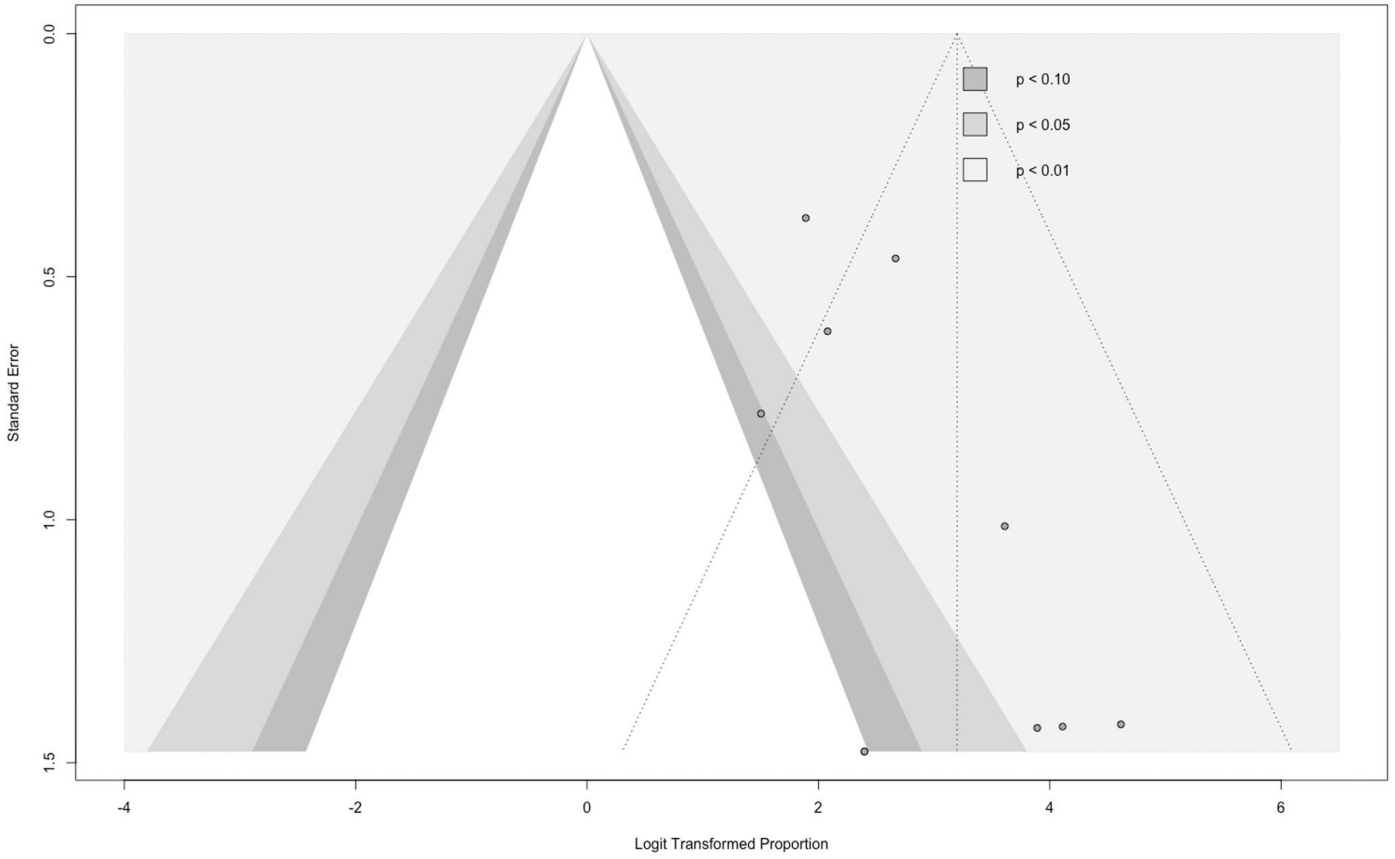
Funnel plot of the included studies.

Although the Egger test did not yield significant results (*P* = 0.05), the possibility of publication bias cannot be ruled out, particularly following an evaluation of the funnel plot in Figure 5. Given the lack of global reporting on TN and FP, calculations for specificity, PPV, and NPV were not achievable.

A sub-analysis including only studies which have used CTP (*n* = 8 studies) showed a combined sensitivity of 96.9 (95% CI, 90.1-99.1).

## Discussion

Our meta-analysis demonstrated that brain perfusion imaging using CT or MR exhibits high sensitivity in the diagnosis of BD, showing comparable, if not superior, performance to CTA^8^ or TCD,^26^ with sensitivity values of 0.84 and 0.90, respectively.

Due to most studies not providing data on TN and FP, it was not feasible to calculate specificity, PPV, and NPV rates.

Traditional neuroimaging methods for determining BD typically rely on assessing brain structures themselves to infer potential lesions. In contrast, perfusion imaging by CT or MR enables a more direct and precise evaluation of brain function, capable of discerning subtle differences^27^ that are critical in the clinical and medicolegal context. This area of research is supported by the World Brain Death Project.^1^

In clinical settings, high-quality diagnostic contrast-enhanced brain perfusion CT and MR imaging must meet specific criteria acquisition and post-processing. The output of most perfusion techniques relies on the arterial input function (AIF) obtained from signal changes in brain vessels post-contrast administration. AIF is determined by placing regions of interest (ROI) on an artery and a high-calibre vein (typically the superior sagittal sinus). Perfusion remains reliable even with different ROI placement for AIF calculation, as observed in clinical scenarios like acute ischaemic stroke.^28^^,^^29^ However, BD is rather different as most if not all vessels usually chosen for AIF are expected to show absent contrast-filling or very slow flow. This could lead to erroneous AIF calculations, resulting in unclear cerebral blood flow (CBF) and cerebral blood volume (CBV) maps. This may have serious consequences. Only 4 out of 9 studies with contrast-based perfusion imaging provided the specific, notwithstanding quite variable, mostly intracranial ROI location for AIF calculation,^16^^,^^19^^,^^23^^,^^25^ (ranging from cavernous internal carotid artery and other intracranial vessels to the superficial temporal arteries). All studies did not report AIF curves or quality control. Ideally, an optimal ROI for AIF should show an early, steep rise from baseline with a narrow peak.^30^ Generally, perfusion imaging in this context could benefit from using ROIs in extracranial structures like the superficial temporal artery. This artery offers less resistance to blood flow, enabling flow even in BD patients. However, this approach lacks validation.

Arterial spin labelling (ASL), a non-contrast-based MR perfusion sequence can also provide perfusion biomarkers based on using different physical principles. Briefly, in its most used and recommended form nowadays,^31^^,^^32^ pseudocontinuous ASL (pcASL) relies on spatially selective magnetization inversion of spins in arterial blood water at the cervical region using radiofrequency (RF) pulses, effectively using blood magnetization as an *arterial spin label*. A specific delay is then allowed to occur, followed by image acquisition distally, at the brain, containing signal from both static tissue water (from intracranial structures which have not moved significantly) and signal from the previously labelled blood molecules. To differentiate between the types of signals, control images in the same planes—without prior arterial spin labelling—are acquired. Calculating signal differences between these images allows estimating the arterial intracranial inflow in absolute units, without the placement of an ROI to calculate AIF as in DSC. The selective labelling using RF pulses in ASL gives it its major advantage over other types of brain perfusion MRI along with the absence of exogenous contrast administration. However, this comes at a cost of a lower signal-to-noise ratio (SNR), the need for higher field strength magnets, and the higher vulnerability of the sequence to motion artefacts.

In ASL, the time between labelling and image acquisition (post-label delay) is crucial. Current clinical practice allows for only one delay, and selecting the best one involves balancing factors like SNR, acquisition time, spatial resolution, and handling specific artefacts. For instance, slow arterial flow (eg, in carotid stenosis) may result in labelled blood molecules not reaching the imaging plane in time, causing errors in CBF calculations unrelated to the true blood flow rate. However, as in other occasions, an unwanted artefact can be made to enhance knowledge and medical care of certain clinical contexts, as has been the case of this “arterial transit artefact” in ASL.^33^^,^^34^

Experts have suggested delays for different medical conditions,^32^ but none has been proposed for assessing BD. Multi-delay implementations of ASL are currently under development to help overcome some of these issues. The only study in our review with ASL^21^ included 5 patients with a single post-label delay of 1.5 s and is therefore not immune to this shortcoming. Nevertheless, this technique holds particular promise, namely in the context of recent contrast shortages,^35^ with the gadolinium-related (known and unknown) risks, and the possibility of accurate, time-efficient measurements with multiple post-label delays.

Another underrecognized source of errors in perfusion CT and MR imaging is related to the possibility of preceded craniotomy. One can expect a 15% reduction in the sensitivity of perfusion imaging (only contrast-based studies have assessed this so far) in patients who underwent craniotomy.^36^ This is similar to what has been described for CTA^37^ and TCD,^38^ translating the compensatory mechanism of an increase in cerebral perfusion pressure when faced with a sudden decrease in intracranial pressure by way of craniectomy.^39^

### Strengths and limitations

A strength of this meta-analysis is the random-effects approach, which accounts for variability between the studies, providing more generalizable results. In addition, the heterogeneity of the studies included was small, reducing concerns about inconsistent study results affecting our conclusions.

The findings of this systematic review and meta-analysis should be interpreted cautiously. The study primarily relies on retrospective research with no control group. Moreover, a number of these studies only presented sensitivity metrics, potentially introducing selection bias. In addition, the definition of BD through brain perfusion was not consistently clear. While 3 groups^19^^,^^20^^,^^24^ used quantitative outcomes—which themselves varied (eg, CBF cut-offs at 10 or 15 mL/100 g/min in different studies, both with similar CBV of 1 mL/100 g)—others either referred to visual assessment of perfusion maps or did not specify further. The different outcomes used by the included studies are available in Table 1.

Furthermore, the timing of perfusion imaging in relation to the determination of BD varied. Some studies did not specify the timing, while others conducted perfusion tests concurrently with clinical assessments or up to 7 days afterwards. Despite these limitations, there is general consensus in the results across different modalities and acquisition settings, including contrast injection rates, with the sensitivity of the index test consistently exceeding 80%.

Beyond the shortcomings of the included studies, the meta-analysis itself has limitations. Firstly, differences in sample sizes could have introduced small-study effects, which may have biased some of the meta-analytic estimates and reduced their precision. However, we managed to replicate our findings in studies with >30 participants. Moreover, publication bias remains a possibility, considering the funnel plot.

This study underscores the critical need for future multicentric prospective studies involving control groups and standardized acquisition protocols.^30^^,^^32^^,^^40^ Collaboration with other professional societies is essential to more precisely define the diagnosis of BD and establish optimal timing for perfusion imaging. Standardization will enhance reproducibility and wider adoption of the technique. Overall, brain perfusion imaging using CT or MRI shows promise in diagnosing BD, emphasizing the importance of continued research in this area.

## Conclusion

CT and MRI brain perfusion imaging demonstrate satisfactory sensitivity, at least equivalent to CTA and TCD, in diagnosing BD. Nevertheless, it is crucial to acknowledge caveats which justify the World Brain Death group’s perspective regarding the applicability of CTP or MRP in this setting. There is a pressing need to pursue more substantial evidence that could lead to impactful changes in clinical practice.

## Supplementary Material

tzae037_Supplementary_Data
